# Supplementary figures and images for: Homeodomain-interacting protein kinase (Hipk) plays roles in nervous system and muscle structure and function
Source: PLoS One. 2020 Mar 18;15(3):e0221006. doi: 10.1371/journal.pone.0221006 (PMC7080231; doi:10.1371/journal.pone.0221006)

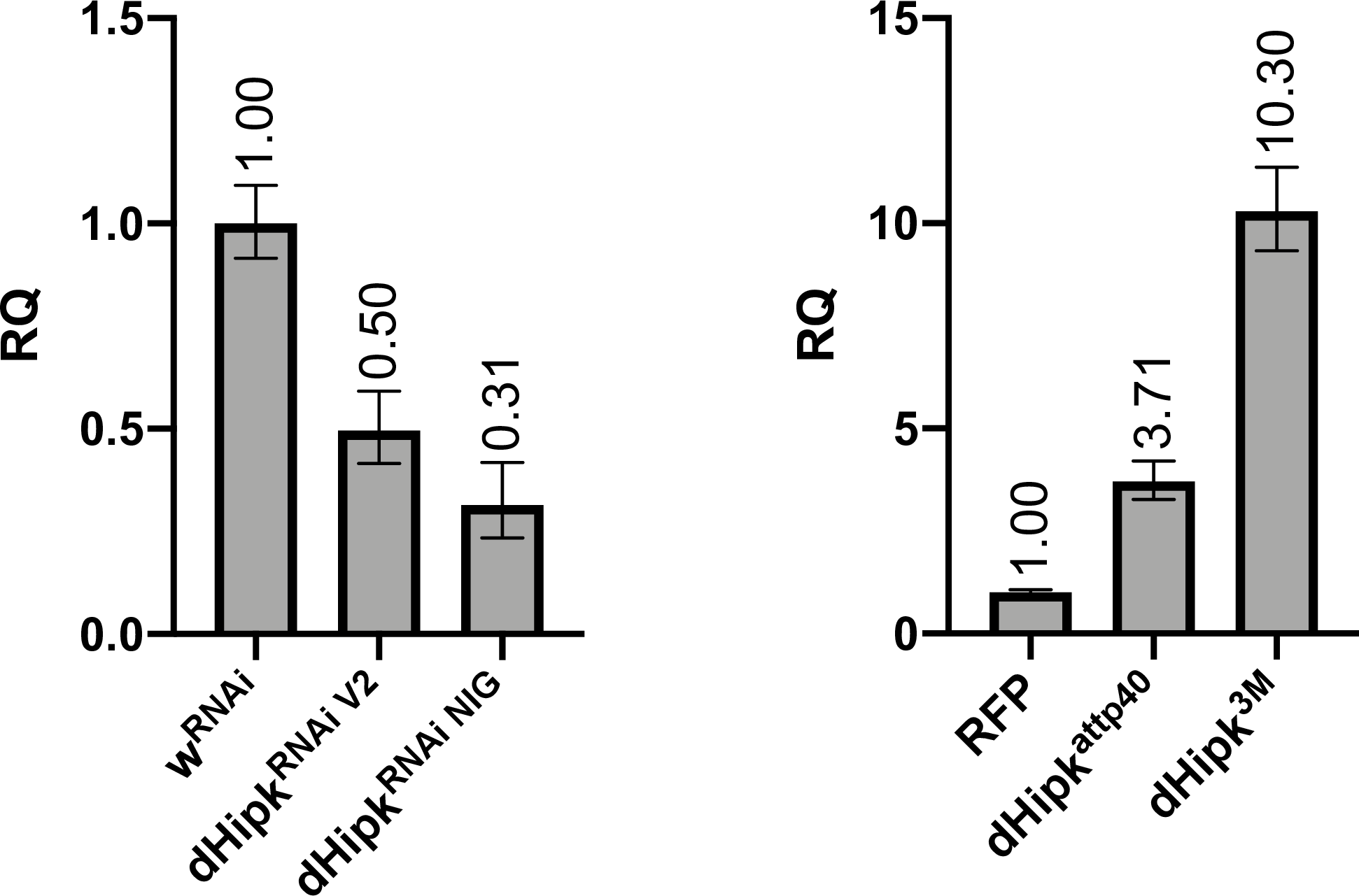

Supplement: S1 Fig — hipk mRNA levels were quantified using qRT-PCR of tissue extracts. (A) Shows relative hipk expression levels following expression of wRNAi (control), dHipkRNAi V2 and dHipkRNAi NIG using Ey-Flp (as described in methods). (B) For quantification of Hipk over-expression, Mef2-Gal4 was crossed to UAS-myr-RFP (control), UAS-Hipk-attp40 and UAS-Hipk3M. All samples were tested in triplicate. Error bars show Min and Max RQ values. (TIF) [file pone.0221006.s001.tif]

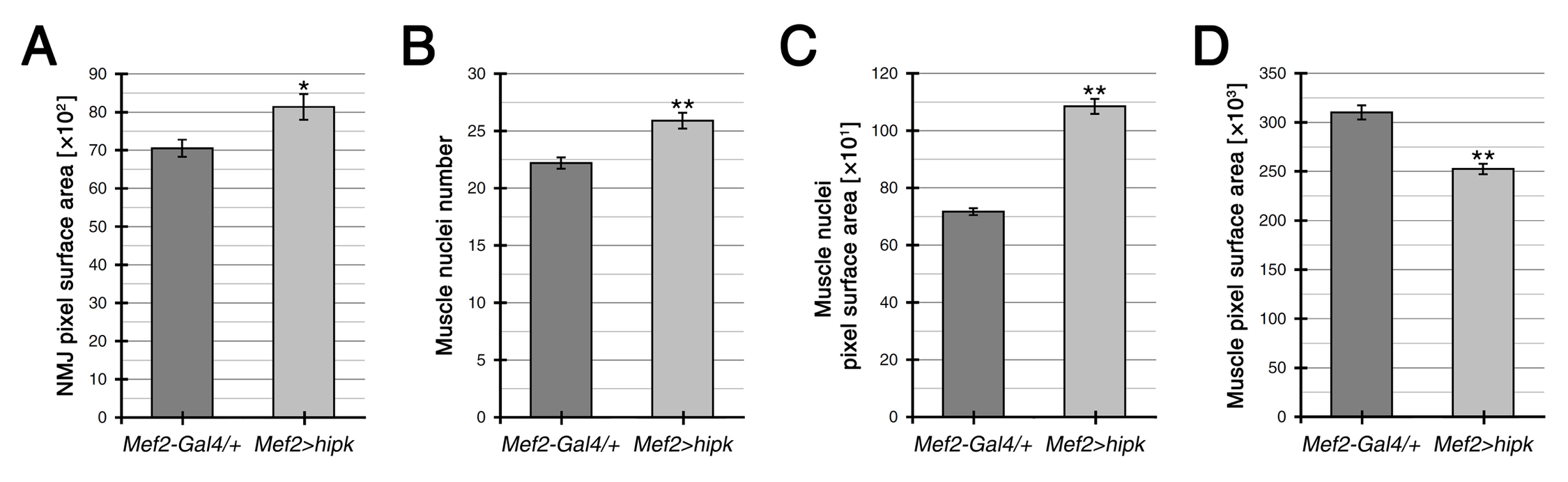

Supplement: S2 Fig — From stacked confocal images of muscles 6/7, NMJs (marked by Hrp), muscle nuclei (marked by DAPI) and muscles were delineated with Photoshop selection tools and their sizes were measured as pixel surface area. (A) Hipk over-expression in the muscle (Mef2-Gal4>hipk) led to significantly larger NMJs (an approximately 1.2 fold increase) when compared to the wild-type control (i.e. Mef2-Gal4 outcrossed to w1118). (B-C) Hipk over-expression also resulted in a slight increase in muscle nuclei (25.9±0.7 nuclei in muscles 6 and 7 combined, in comparison to 22.2±0.5 in the wild-type control), which were significantly bigger on average (an approximately 1.5 fold increase). (D) Quantification shows that Hipk over-expressors had significantly smaller muscles (an approximately 1.2 fold decrease), indicating that the measured Hipk-mediated effects on NMJ (A) and muscle nuclei (C) size shown here are not simply due to increased muscle size. Sample size was 24 muscle 6/7 pairs from 12 body walls for each genotype. * p = 0.0106; ** p < 0.0001. (TIF) [file pone.0221006.s002.tif]

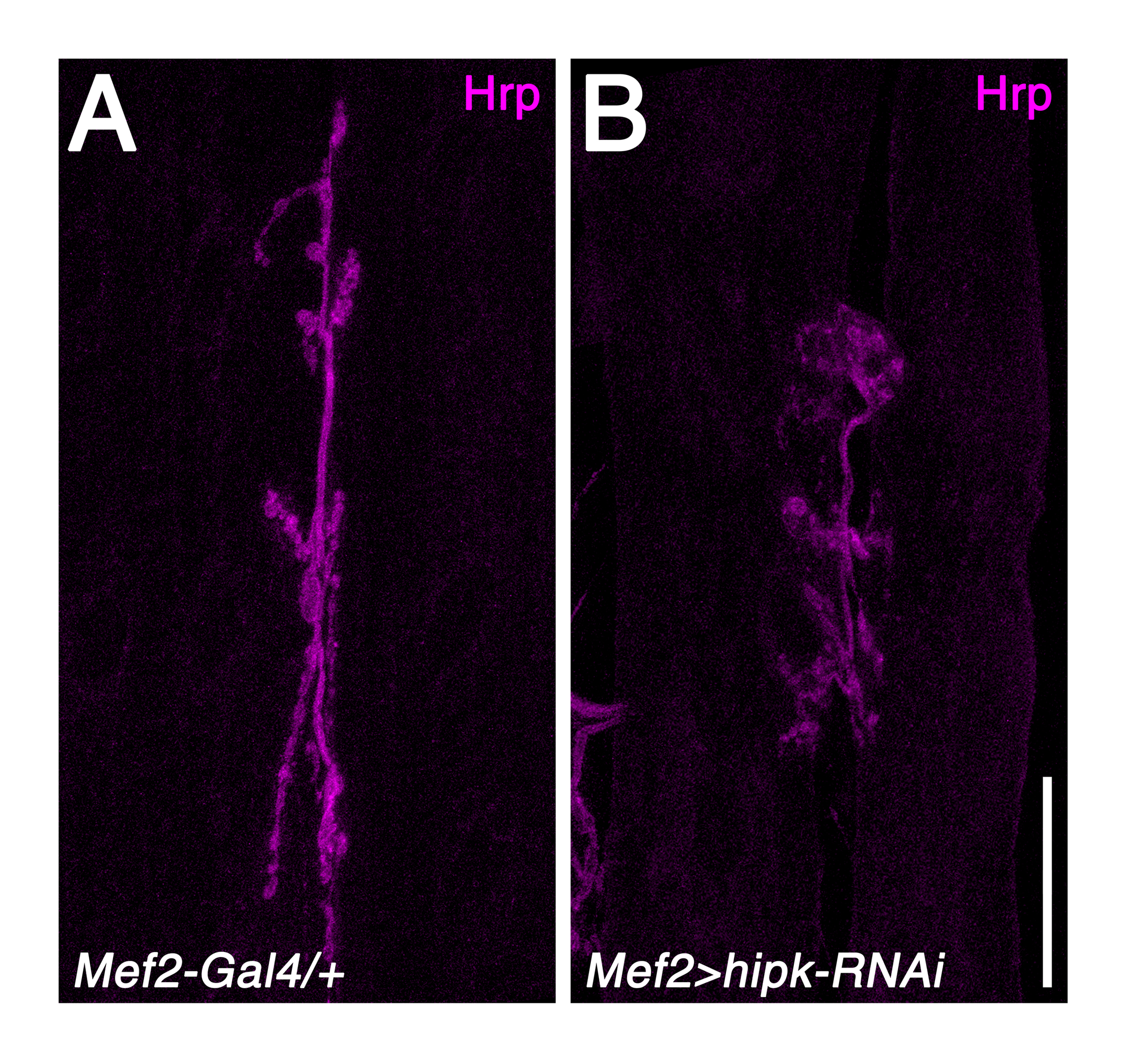

Supplement: S3 Fig — (A) A typical 3rd instar larval NMJ, marked by Hrp, innervating muscles 6/7 in abdominal segment 3. (B) Muscle-specific expression of transgenic hipk-RNAi (Mef2-Gal4>hipk-RNAi) resulted in NMJ (note the slightly diffuse Hrp labeling) and muscle morphological defects. Scale bar: 40μm (A,B). (TIF) [file pone.0221006.s003.tif]

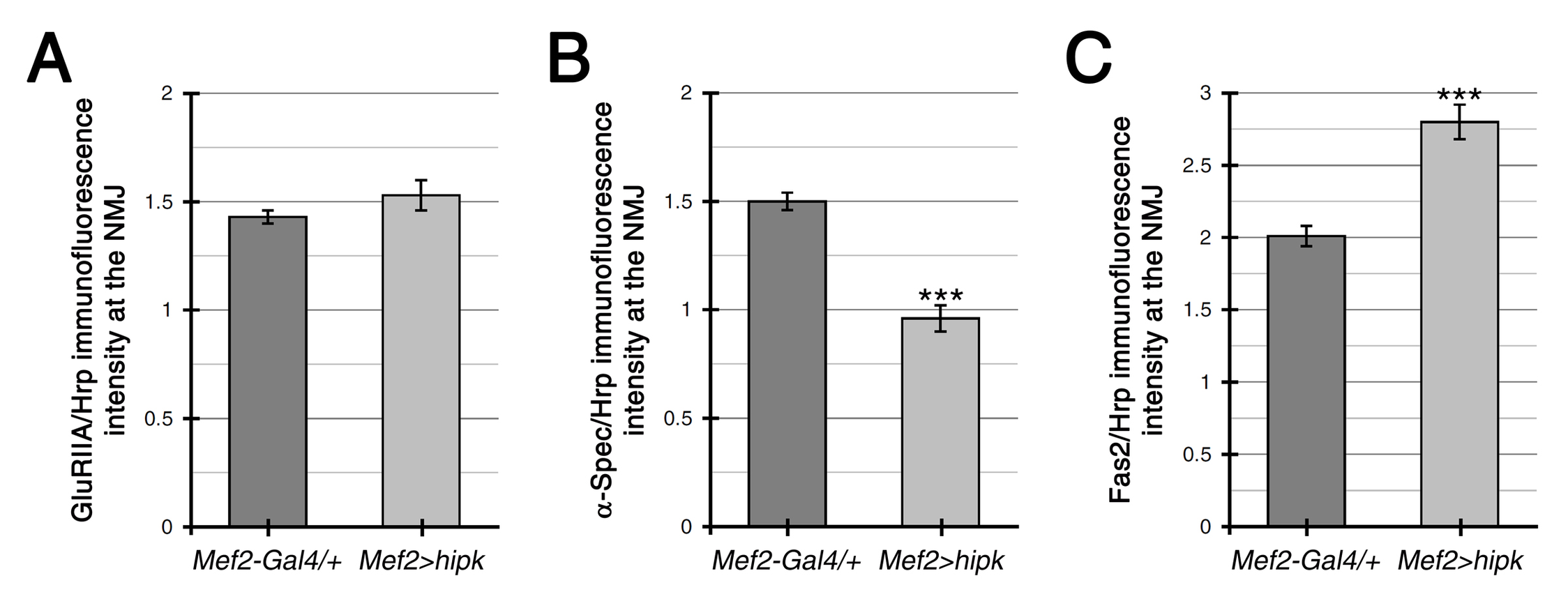

Supplement: S4 Fig — Post-synaptic protein levels at the NMJ was calculated as a ratio between the immunofluorescence intensity of the protein of interest and Hrp, which was used as a staining control. Immunofluorescence signal at the NMJ was selected using Photoshop, and the intensity was determined by measuring the mean gray value. (A) No significant effects on the levels of GluRIIA at the NMJ were observed between muscle-specific Hipk over-expression and the control. n = 8 (over 4 body walls) for each genotype. (B) However, Hipk over-expression resulted in significantly lower synaptic levels of α-Spec. n = 8 (over 4 body walls) for each genotype. (D) Hipk over-expression also resulted in significantly higher Fas2 synaptic levels. n = 8 (over 4 body walls) for each genotype. *** p < 0.0001. (TIF) [file pone.0221006.s004.tif]

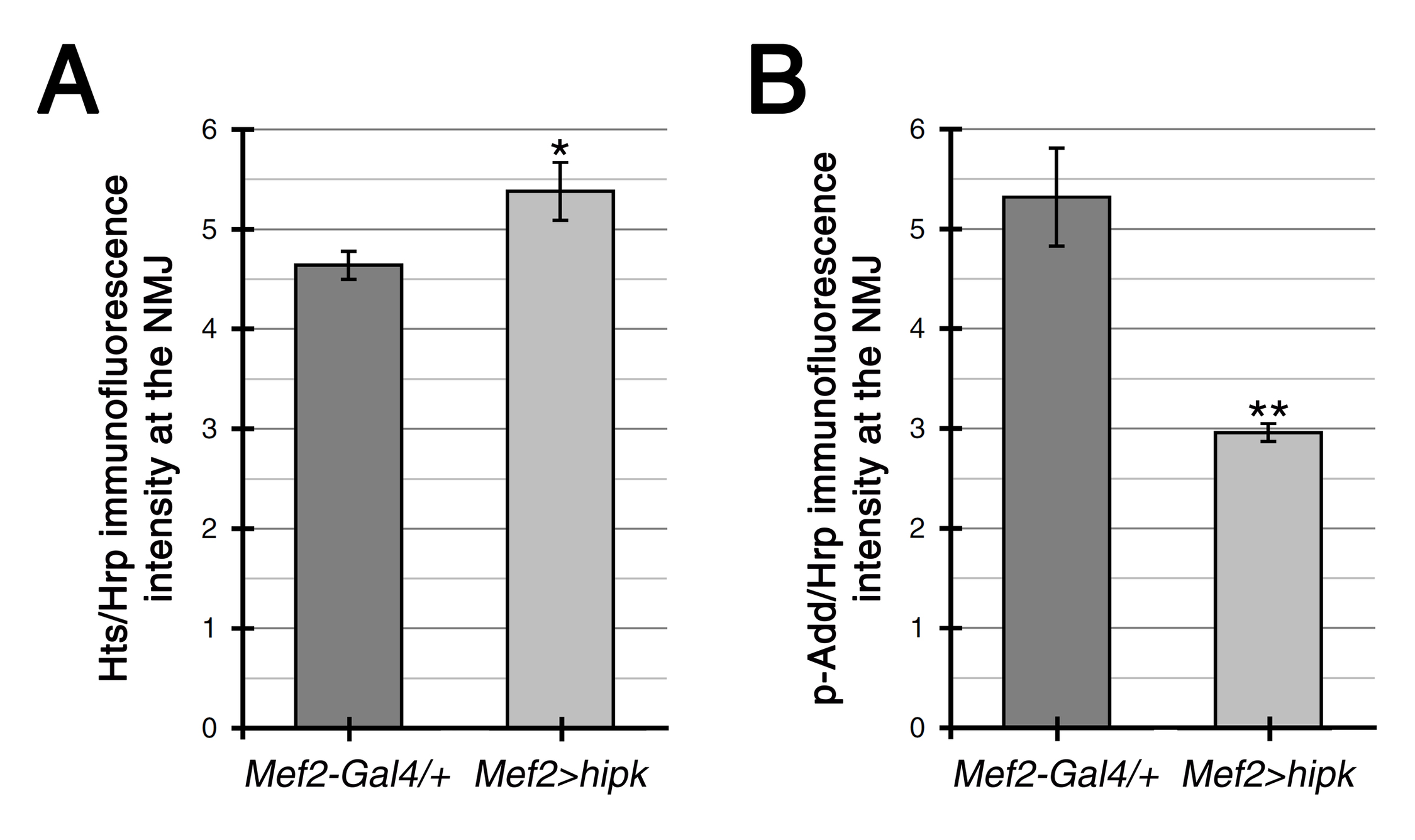

Supplement: S5 Fig — Hts total and phosphorylation levels at the NMJ were calculated as a ratio between Hts or p-Add immunofluorescence intensity and Hrp, which was used as a staining control. Immunofluorescence signal at the NMJ was selected using Photoshop, and the intensity was determined by measuring the mean gray value. (A) Hipk over-expression in the muscle resulted in a slight, but significant, increase in Hts levels at the NMJ when compared to control. n = 8 (over 4 body walls) for each genotype. (B) In contrast, Hipk over-expression led to a significant decrease in p-Add levels at the NMJ. n = 8 (over 4 body walls) for each genotype. * p = 0.0375; ** p = 0.0007. (TIF) [file pone.0221006.s005.tif]

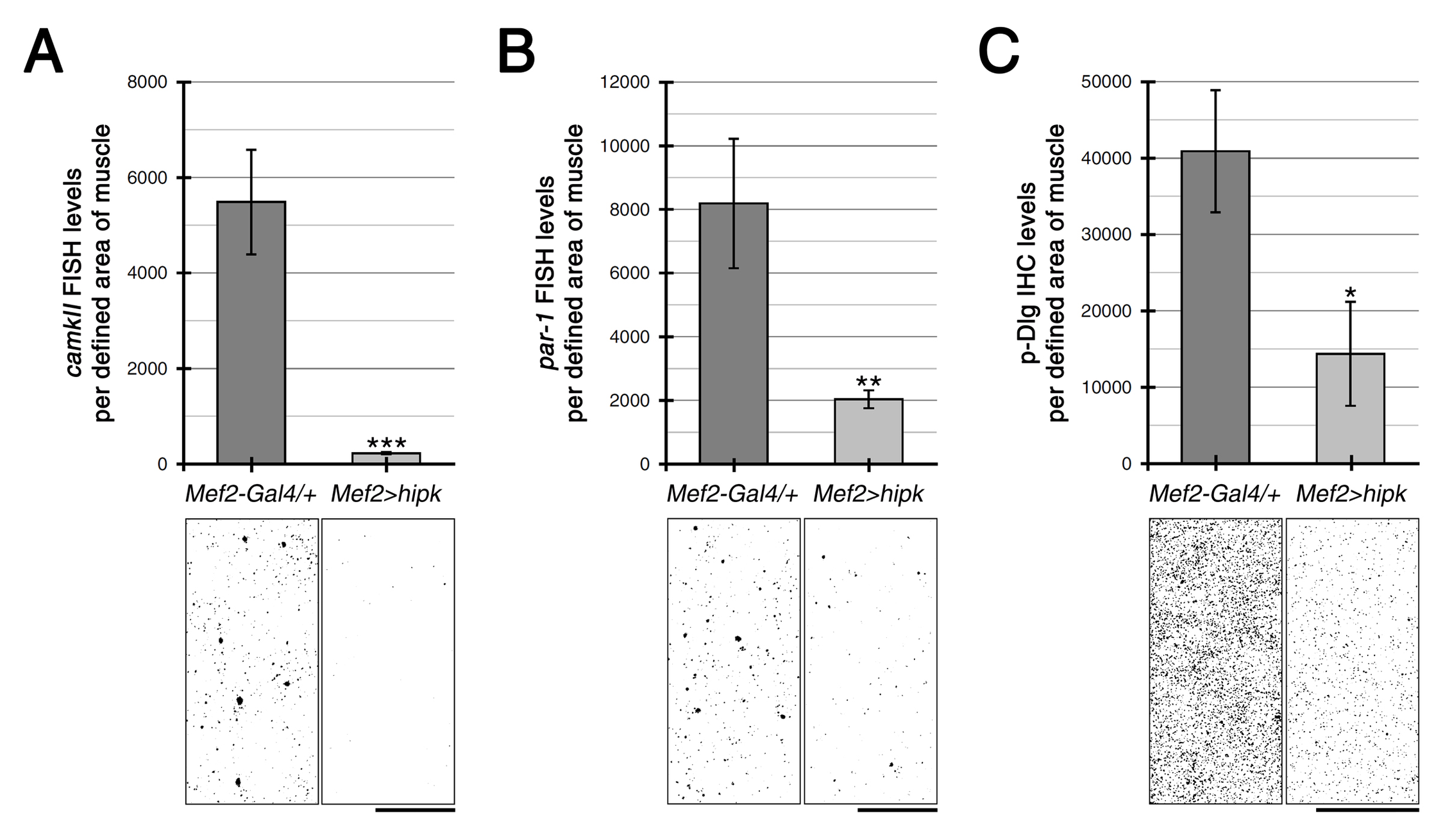

Supplement: S6 Fig — Confocal images were converted to binary images, where immunofluorescent puncta were represented as black pixels and the background was represented as white pixels (examples are shown). The number of extra-synaptic black pixels within a fixed area size of muscle was measured. (A) Muscle-specific Hipk over-expression led to significantly reduced levels of camkII FISH signal when compared to the control. n = 20 (over 5 body walls) for each genotype. (B) Similar results were observed for par-1 FISH signal. n = 16 (over 4 body walls) for each genotype. (C) p-Dlg IHC signal in the muscle was also significantly lowered when Hipk was over-expressed in the muscle. n = 16 (over 4 body walls) for each genotype. * p = 0.019; ** p = 0.0007; *** p < 0.0001. Scale bars: 40μm (A); 40μm (B); 40μm (C). (TIF) [file pone.0221006.s006.tif]

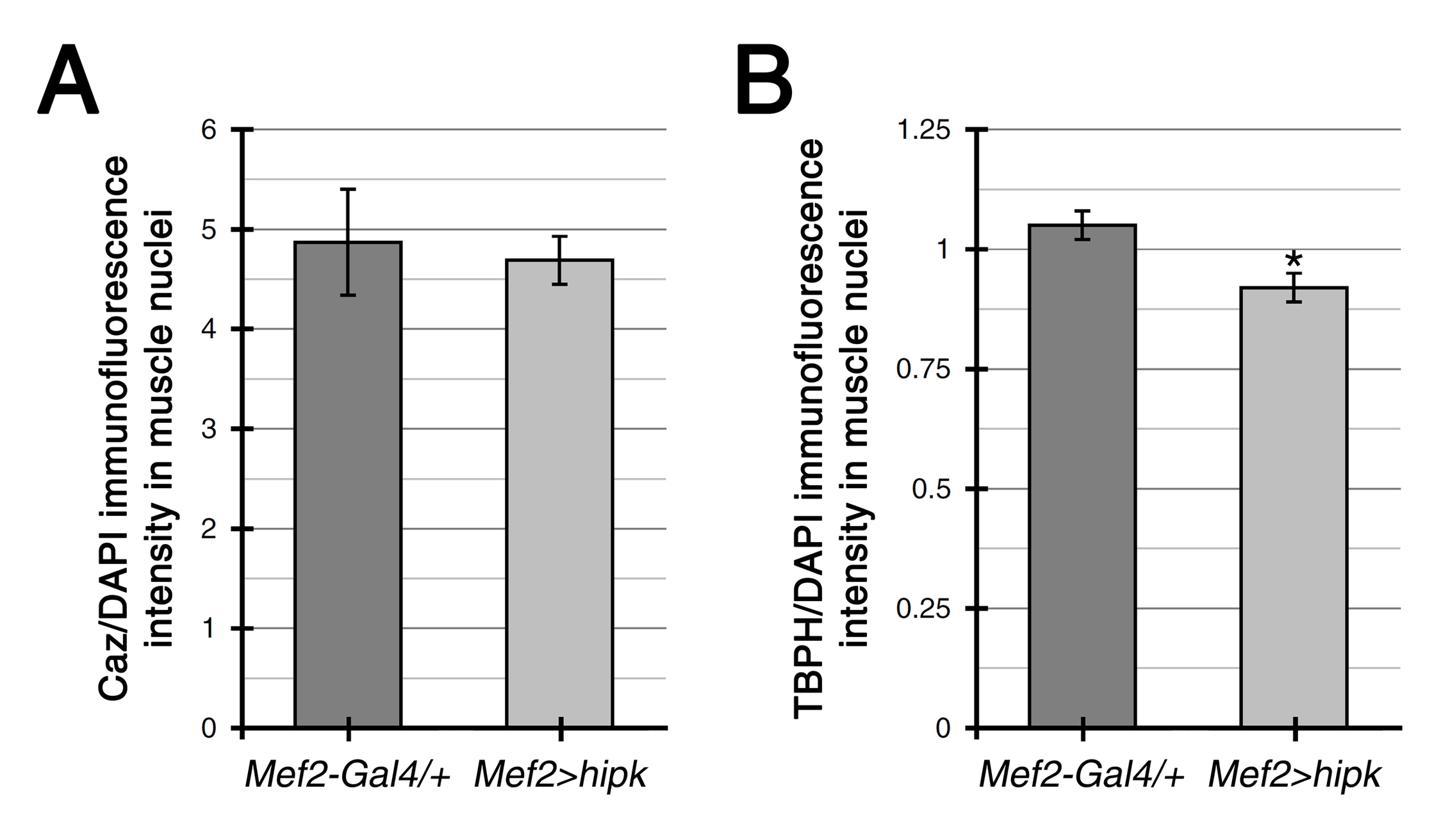

Supplement: S7 Fig — Muscle nuclear levels were calculated as a ratio between either Caz or TBPH immunofluorescence intensity and DAPI, which was used as a staining control. Immunofluorescence signal in muscle nuclei was selected using Photoshop, and the intensity was determined by measuring the mean gray value. (A) No significant effects on the nuclear levels of Caz were observed between muscle-specific Hipk over-expression and the control. n = 10 (over 5 body walls) for each genotype. (B) However, Hipk over-expression resulted in a significant decrease in TBPH nuclear levels when compared to the wild-type control. n = 10 (over 5 body walls) for each genotype. * p = 0.0338. (TIF) [file pone.0221006.s007.tif]
