# Supplementary material for: Homeodomain-interacting protein kinase (Hipk) plays roles in nervous system and muscle structure and function
Source: PLoS One. 2020 Mar 18;15(3):e0221006. doi: 10.1371/journal.pone.0221006 (PMC7080231; doi:10.1371/journal.pone.0221006)
Supplement: S1 Table — (DOCX) [file pone.0221006.s008.docx]

| *Gal4* Driver | Expression | % Progeny | | Total Flies | Comments on  *UAS-hipk-RNAi* |
| --- | --- | --- | --- | --- | --- |
|  |  | *UAS-hipk-RNAi* | Control (Balancer) |  |  |
| *Appl** | Pan-neuronal | 9.1 (M) | 90.9 (M) | 44 | Male semi-lethal |
|  |  | 56.7 (F) | 43.3 (F) | 180 | Viable |
| *Appl*** | Pan-neuronal | 45.8 (F) | 54.2 (F) | 72 | Viable |
| *ple* | Dopaminergic | 64.7 | 35.3 | 190 | Viable |
| *TH* | Dopaminergic | 40.9 | 59.1 | 154 | Viable |
| *repo* | Glial cells | 64.6 | 35.4 | 130 | Viable |
| *Mef2* | Muscle | 0 | 100 | 162 | Lethal |

Supplementary Table 1: Tests for viability of *hipk* knockdown in various nervous system components and muscles using the VDRC *UAS-hipk-RNAi*
