# Supplementary material for: Homeodomain-interacting protein kinase (Hipk) plays roles in nervous system and muscle structure and function
Source: PLoS One. 2020 Mar 18;15(3):e0221006. doi: 10.1371/journal.pone.0221006 (PMC7080231; doi:10.1371/journal.pone.0221006)
Supplement: S2 Table — (DOCX) [file pone.0221006.s009.docx]

| *Gal4* Driver | Expression | % Progeny | | Total Flies | Comments on  *UAS-HA-hipk* |
| --- | --- | --- | --- | --- | --- |
|  |  | *UAS-HA-hipk* | Control (Balancer) |  |  |
| *Appl** | Pan-neuronal | 2.9 (M) | 97.1 (M) | 34 | Lethal |
|  |  | 7.5 (F) | 92.5 (F) | 106 | Semi-lethal |
| *Appl*** | Pan-neuronal | 14.3 (F) | 85.7 (F) | 49 | Semi-lethal |
| *ple* | Dopaminergic | 49.6 (M) | 50.4 (M) | 123 | Viable |
| *TH* | Dopaminergic | 19.3 (M) | 80.7 (M) | 83 | Male semi-lethal  (abnormal pigmentation) |
|  |  | 48.1 (F) | 51.9 (F) | 154 | Viable |
| *repo* | Glial cells | 39.5 | 60.5 | 248 | Viable |
| *Mef2* | Muscle | 57.2 | 42.8 | 257 | Viable |

Supplementary Table 2: Tests for viability of *hipk* over-expression in various nervous system components and muscles using the attP40 *UAS-HA-hipk*

**Gal4* females used in cross; ***Gal4* males used in cross; (M) = Males only; (F) = Females only
